# Canonical Correlation of Milk Composition Parameters and Blood Biomarkers in High-Producing Dairy Cows During Different Lactation Stages

**DOI:** 10.3390/ani14223294

**Published:** 2024-11-15

**Authors:** Eva Kovacikova, Anton Kovacik, Lubos Harangozo, Katarina Tokarova, Zuzana Knazicka, Eva Tvrda, Tomas Jambor, Marian Tomka, Peter Massanyi, Norbert Lukac

**Affiliations:** 1 Institute of Nutrition and Genomics, Faculty of Agrobiology and Food Resources, Slovak University of Agriculture in Nitra, Tr. A. Hlinku 2, 949 76 Nitra, Slovakia; eva.kovacikova@uniag.sk (E.K.); zuzana.knazicka@uniag.sk (Z.K.); 2 AgroBioTech Research Centre, Slovak University of Agriculture in Nitra, Tr. A. Hlinku 2, 949 76 Nitra, Slovakia; 3 Institute of Applied Biology, Faculty of Biotechnology and Food Sciences, Slovak University of Agriculture in Nitra, Tr. A. Hlinku 2, 949 76 Nitra, Slovakia; katarina.tokarova@uniag.sk (K.T.); tomas.jambor@uniag.sk (T.J.); peter.massanyi@uniag.sk (P.M.); norbert.lukac@uniag.sk (N.L.); 4 Institute of Food Sciences, Faculty of Biotechnology and Food Sciences, Slovak University of Agriculture in Nitra, Tr. A. Hlinku 2, 949 76 Nitra, Slovakia; lubos.harangozo@uniag.sk; 5 Institute of Biotechnology, Faculty of Biotechnology and Food Sciences, Slovak University of Agriculture in Nitra, Tr. A. Hlinku 2, 949 76 Nitra, Slovakia; eva.tvrda@uniag.sk (E.T.); marian.tomka@uniag.sk (M.T.); 6 Institute of Biology, Faculty of Exact and Natural Sciences, University of the National Education Commission, ul. Podchorążych 2, 30-084 Krakow, Poland

**Keywords:** blood biomarkers, milk composition, health status, lactation stage, cattle, canonical correlation

## Abstract

This study explores milk composition and blood markers in cows across lactation stages. Holstein cows were divided into four groups: beginning of lactation (BL; *n* = 21), peak of lactation (PL; *n* = 21), middle of lactation (ML; *n* = 21), and end of lactation (EL; *n* = 20). Blood (1 × 15 mL) and milk samples (1 × 100 mL) were collected for biomarker analysis. Blood chemistry profiles were determined using a clinical chemistry analyser, and milk lactose, fat, and protein levels (%) were determined using an infrared absorbance analyser. Minerals (Ca, P, and Mg) in milk were determined by atomic absorption spectrometry after mineralizing the samples. Glucose was higher in the EL group than in the BL group (*p* < 0.01), whereas D-beta-hydroxybutyrate (D-BHB) was higher in the BL group than in the PL and ML groups (*p* < 0.001). Cholesterol was higher in the PL, ML, and EL groups than in the BL group (*p* < 0.001). Gamma-glutamyl transferase was increased in the PL group compared to the BL group. Phosphorus levels were lower in the PL than in the BL group, whereas protein levels were higher in the EL than in the PL group. Spearman and partial correlation analysis showed several significant associations between the observed variables. Using canonical correlation analysis were identified three significant correlations (*r*_*c*1_ = 0.853; *r*_*c*2_ = 0.823; *r*_*c*3_ = 0.739). The main canonical correlation identified blood TG and milk urea as the strongest variables. According to the canonical loading, the biomarkers TG, Mg, urea, cholesterol, and alkaline phosphatase (*U1*) are the primary variables associated with milk parameters (*V1*), specifically with milk urea, milk Mg and P, protein, and lactose.

## Simple Summary

Key findings of our study included strong positive relationships between lactation stages and blood biomarkers like glucose and cholesterol, as well as milk protein levels. Blood biomarkers such as triglycerides, magnesium, and urea showed significant correlations with various milk parameters. Canonical correlation analysis identified strong relationships between blood biomarkers and milk composition, with blood triglycerides and milk urea being the most influential variables. The presented findings suggest that understanding these correlations can help improve dairy cow management and milk production strategies.

## 1. Introduction

Cows adapt differently to lactation and may, therefore, show distinct changes in milk composition and susceptibility to production-related diseases as they pass through various lactation stages [1]. The most critical period in the productive life of high-yielding dairy cows is the transition period [2]. During this period, metabolic disorders are a major problem. These disorders mainly occur due to difficulties in adapting to various internal and external changes and due to deficits in nutrient supply relative to demand [3]. The sudden high nutrient demand for milk production after parturition exceeds the cow’s adaptive capacity.

Common metabolic disorders resulting from nutrient deficits include ketosis, milk fever, and negative energy balance (NEB) [4,5,6,7,8]. Ketosis in dairy cattle primarily occurs between 2 and 7 weeks postpartum [9,10]. However, diagnosing metabolic disorders can be challenging. To address this issue, several detection methods have been developed. One of the most important methods is the metabolic profile test [11,12,13]. It enables the detection of metabolic disorders in preclinical stages, even before the clinical signs appear [14]. Beta-hydroxybutyrate (BHB) levels in both blood and milk correlate and are effective indicators of subclinical ketosis (SCK) in dairy cows [15]. Due to BHB’s positive correlation with the degree of negative energy balance, the fat-to-protein ratio (F:P ratio) has been proposed as an alternative method to diagnose SCK [16,17,18]. This ratio is calculated by dividing the fat content of milk by its protein content Unlike fat and protein content, which can be diluted as milk yield increases, the F:P ratio remains relatively independent of milk quantity, making it a better indicator of energy balance [16,19].

Milk contains various markers/factors that are indicative of dairy cows’ metabolic health. Changes in the abundance of these substances, which thereby affects milk composition [20,21], can negatively affect the quality of milk products. According to Bland et al. [22], fat, lactose, and urea are the main factors influencing milk coagulation and coagulum properties. Milk quality is affected by several factors, such as nutrition, genetics, and breeding management [23,24,25,26,27,28,29]. Mineral content and calcium-to-phosphorus ratio are also important parameters that determine the quality of milk with regard to human nutrition. Increasing dietary phosphorus raises serum phosphorus levels, which temporarily lowers serum ionized calcium. This can lead to elevated parathyroid hormone secretion and potential bone resorption [30]. Early diagnosis and preventive measures could prevent the occurrence of clinical symptoms and hence their impact on the farming economy.

In recent decades, dairy farmers have prioritized high milk production for economic reasons. Consequently, traditional breeds across Europe are being replaced by the Holstein breed. This push for higher yields places a significant burden on animal health and metabolic status. Therefore, monitoring the health of high-producing animals remains crucial. Additionally, ensuring high milk quality is important for processing and consumers. Several studies have explored changes in milk production and composition, particularly during the transition [10] and early lactation periods [31] or across all lactation stages [32].

In our study, the objective was to compare the serum biochemical parameters and basic milk composition in high-yielding dairy cows during different lactation stages. We also aimed to identify the correlations between serum and milk parameters. We hypothesized that there are significant differences between serum biochemical parameters and milk composition that vary across different lactation stages. By studying these relationships, biomarkers/factors affecting the milk quality could be revealed. This ongoing monitoring is important for managing animal health throughout the entire lactation period.

## 2. Material and Methods

### 2.1. Animals

The samples for this study were collected from December to March (120 days) at the university farm in Oponice (VPP SPU Ltd., Oponice, Slovak Republic), where dairy cows were housed in free-stall barns. In total, 83 Holstein Friesian dairy cows (in the second lactation) were selected for analysis and were divided into four groups based on lactation stage: beginning of lactation (BL; *n* = 21; 14–49 days of lactation), peak of lactation (PL; *n* = 21; 50–109 days of lactation), middle of lactation (ML; *n* = 21; 110–209 days of lactation), and end of lactation (EL; *n* = 20; 210–305 days of lactation). The average milk yield at a particular farm was 9994 litres per lactation (305 days) per dairy cow.

The cows were fed a total mixed ration (TMR) twice daily and had ad libitum access to water, following National Research Council (NRC) standards [33]. The feed and nutrient composition are detailed in Table 1. The analysis of organic and inorganic nutrient content followed previous studies [34,35]. No clinical signs of metabolic or inflammatory disease were observed in any of the dairy cows during the study.

### 2.2. Blood Sampling and Analysis

Blood samples (1 × 15 mL) for biochemical analyses were collected two h after the morning feeding through *vena caudalis mediana* puncture. Samples were collected directly into centrifuge tubes, and serum was separated through centrifugation at 1006× *g* and 20 °C for 20 min. Sampling took place in three periods (during winter and early spring), and in each period, samples were taken from all groups. Collections were always planned +/− seven days in the middle of a specific lactation period based on information from the university farm.

The following profiles were assessed using blood serum: energy [glucose (GLU), D-beta-hydroxybutyrate (D-BHB), and triglycerides (TG)], nitrogen [total protein (TP) and urea], hepatic [aspartate aminotransferase (AST), alanine aminotransferase (ALT), gamma-glutamyl transferase (GGT), alkaline phosphatase (ALP), bilirubin (BILI), and cholesterol (CHOL)], and mineral [calcium (Ca), phosphorus (P), and magnesium (Mg)].

All analyses were performed in duplicates using commercial kits (DiaSys, Diagnostic Systems GmbH, Holzheim, Germany; Randox Laboratories Ltd., Crumlin, UK) and an Rx Monza clinical chemistry analyser (Randox Laboratories Ltd.) [36,37]. Intra- and inter-assay coefficients and sensitivities for the selected parameters are detailed in Table 2.

### 2.3. Milk Sampling and Analysis

Samples (1 × 100 mL) were collected during morning milking using a DeLaval AB MM6 milk meter (DeLaval, Tumba, Sweden) and stored at 6 °C. Lactose, fat, and protein levels (%) in whole milk were determined using an infrared absorbance analyser (Milkoscan™ FT 120, FOSS, Hilleroed, Denmark). Milk fat to milk protein ratio (F:P ratio) was calculated by dividing total fat content by total protein content.

To obtain the milk supernatant, 0.1 mL of 10% sulfuric acid was added to 5 mL of the milk sample, followed by mixing and centrifugation (10 min at 2000 rpm; parameters optimized by the authors). Urea levels were assessed from the supernatant using a commercial kit (DiaSys; Diagnostic Systems GmbH) and an Rx Monza clinical chemistry analyser (Randox Laboratories Ltd.).

Mineral contents (Ca, P, and Mg) were determined by atomic absorption spectrometry after mineralizing the samples. Briefly, 10 mL of concentrated nitric acid and 5 mL of concentrated perchloric acid were added to 5 mL of milk sample. After 24 h, all the samples were heated in a sand bath and filtered through a filter paper (84 g/m^2^) (method optimized by the authors). All the measurements were provided in duplicates.

### 2.4. Data Management and Statistical Analysis

GraphPad Prism (version 8.0.1.; GraphPad Software, Boston, MA, USA) was used to conduct statistical analyses. The data sets were subjected to the Shapiro-Wilk test for normal distribution and are presented as the mean ± standard error of the mean (SEM). The one-way analysis of variance (ANOVA) with post hoc Tukey’s multiple comparison tests was used to assess differences between groups if they passed the normality test. If they did not pass the normality test, we used the non-parametric Kruskal-Wallis test with Dunn’s multiple comparisons. Spearman rank-correlation (crude and partial) coefficients (*r*), adjusted for the lactation stage, were used to assess associations between serum biomarkers and milk composition parameters. The significance level was set at *p* < 0.05. The canonical correlation analysis (CCA) was used for the exploration of correlations between the linear combinations of two sets of variables (first set of variables *Ui* = blood biomarkers versus second set of variables *Vj* = milk composition parameters; *i* = 14 dependent variables; *j* = 8 independent variables). Correlation analyses were analysed using the Statgraphic Centurion (version 19; Statgraphics Technologies, Inc. The Plains, VA, USA) and OriginPro 2024b 10.15 (OriginLab Corporation, Northampton, MA, USA).

## 3. Results

### 3.1. Serum Biochemistry and Milk Composition

The results of serum and milk biochemical analyses are summarized in Table 3. Serum Ca levels were non significantly lower in the BL, PL, and ML groups compared to the EL period. There was a significant difference in serum magnesium levels between the PL and EL groups (*p* = 0.016). While serum urea level was lowest in the BL group, total protein levels were higher in the PL, ML, and EL groups. However, these differences were not significant. A significant increase in the GLU levels was observed in the EL group compared to the BL group (*p* = 0.004). In contrast, D-BHB levels were significantly higher in the BL group than in all other groups (*p* = 0.002).

In terms of variance during lactation stages, the hepatic profile exhibited the greatest differences. The serum cholesterol level was within the normal range only in the BL group, while the other groups showed a significant increase. In addition, elevated AST activity was found in the BL, PL, and EL groups, whereas elevated ALT activity was observed only in the PL group (however, both non-significantly). GGT activity was elevated across all groups, with a significant difference between the PL and BL groups (*p* = 0.036).

The analysis of milk composition showed significant differences based on the lactation stage. Phosphorus levels were significantly higher in the EL group compared to the PL and ML group (*p* = 0.004). Milk protein content was significantly higher in the EL group than in all other groups (*p* = 0.016). Additionally, milk yield varied by lactation stage, with significant differences between the PL group and all other stages (BL, ML, and EL). The levels of milk Ca, Mg, lactose, and urea were balanced across all lactation stages.

### 3.2. Correlations Between Blood Markers and Milk Composition

Firstly, the results of correlation analysis (Spearman) are presented in Table 4. There was a high positive significant correlation between lactation stage and blood serum markers, especially P (*r* = 0.2209), GLU (*r* = 0.3375), and CHOL (*r* = 0.2834). A positive significant relationship between lactation stage and milk protein levels (*r* = 0.4209) was also observed. Milk Ca correlated with blood minerals, Ca (*r* = 0.2462), P (*r* = −0.5897), and Mg (*r* = 0.2990), as well as ALT (*r* = 0.4896) and TG (*r* = −0.3769). Statistically significant correlations were observed between milk P and blood P (*r* = 0.3905), Mg (*r* = −0.4156), UREA (*r* = −0.2967), TP (*r* = −0.2394), ALT (*r* = −0.2965), ALP (*r* = 0.3140), and CHOL (*r* = −0.2708). The last observed mineral (Mg) significantly correlated with blood Ca (*r* = −0.3173), Mg (*r* = 0.4219), D-BHB (*r* = −0.2508), and TG (*r* = 0.3161). Milk fat levels significantly correlated with blood Mg (*r* = −0.2503), GGT (*r* = −0.2538), and CHOL (*r* = −0.2419), while milk protein was significantly correlated only with Mg (*r* = −0.3130) among the blood parameters. Moreover, lactose levels were correlated with blood Ca (*r* = 0.2236), P (*r* = 0.2411), GGT (*r* = 0.2866), and CHOL (*r* = 0.4424), while milk urea levels were correlated with blood Ca (*r* = 0.2255), P (*r* = −0.4444), Urea (*r* = 0.5643), ALT (*r* = 0.4054), ALP (*r* = −0.3740), BILI (*r* = −0.2277) and TG (*r* = −0.4667). Finally, a significant correlation was found between the F:P ratio and blood TP (*r* = 0.2518), CHOL (*r* = 0.2177), and D-BHB (*r* = 0.2344).

Secondly, as shown in Table 5, partial correlation coefficients for the variables of milk composition and blood biomarkers also displayed some important associations/relationships. Positive correlations were found between milk Ca and blood Ca (*r* = 0.3713); milk P and blood P (*r* = 0.2871); milk Mg and blood Mg (*r* = 0.5316); milk Mg and TG (*r* = 0.3756); lactose and CHOL (*r* = 0.3445); lactose and TG (*r* = 0.2820); milk urea and blood urea (*r* = 0.4569); and between F:P ratio and blood P (*r* = 0.2735). Negative correlations were found between milk Ca and blood P (*r* = −0.4620); milk P and blood Mg (*r* = −0.4030), CHOL (*r* = −0.3327), TG (*r* = −0.3478); milk fat and blood P (*r* = −0.3220); lactose and blood P (*r* = −0.3324); and between milk urea and ALP (*r* = −0.2997).

Thirdly, testing of the multidimensional relationship defined eight canonical correlations with three significant canonical functions (Table 6) for the association of canonical variables in individual sets. The conducted CCA between the variables for blood and milk confirmed the first canonical correlation coefficient *r*_*c*1_ = 0.853, indicating a strong correlation between the linear combination of blood biomarkers and the tested composition of milk (*p* = 0.000). Based on this, we can speak of a strong statistically significant relationship between these groups of variables, with the strongest variable being TG and milk UREA. Thus, the first set of linear combinations were *U1* = 0.030076×Ca + 0.050478×P − 0.52053×Mg + 0.33016×Urea + 0.10597×TP + 0.097525×Glu − 0.037887×AST + 0.19245×ALT + 0.0017959×GGT − 0.26182×ALP − 0.28326×Chol. + 0.12774×BHB + 0.13508×Bili − 0.59207×TG and *V1* = 0.1588×Ca (M) + 0.37466×P (M) − 0.675×Mg (M) + 0.08815×Fat (M) + 0.28031×Protein (M) − 0.24403×Lactose (M) + 0.74752×UREA (M) − 0.23417×F:P ratio (“M” means milk parameter), where the variables have been standardized and divided by their standard deviations.

The second canonical correlation coefficient with a value of *r*_*c*2_ = 0.823 also confirms a highly significant relationship of the canonical variables (*p* = 0.000), with greater weights for the variables ALT, UREA, and CHOL. The second set of linear combinations were *U2* = −0.17479×Ca − 0.24271×P − 0.23944×Mg + 0.32338×Urea − 0.10666×TP − 0.15802×Glu − 0.083501×AST + 0.4291×ALT + 0.053097×GGT − 0.23556×ALP − 0.29207×Chol. − 0.14018×BHB − 0.04763×Bili + 0.15909×TG and *V2* = − 0.015284×Ca (M) − 0.63395×P (M) + 0.49668×Mg (M) + 0.47801×Fat (M) − 0.051133×Protein (M) + 0.33808×Lactose (M) + 0.55078×UREA (M) − 0.21569×F:P ratio (“M” means milk parameter).

The third standardized canonical correlation was the last statistically significant one, with *r*_*c*3_ = 0.739 and *p* = 0.002. Here, the weights of the individual variables were spread across a larger number of variables (data not presented).

**Figure 1:**
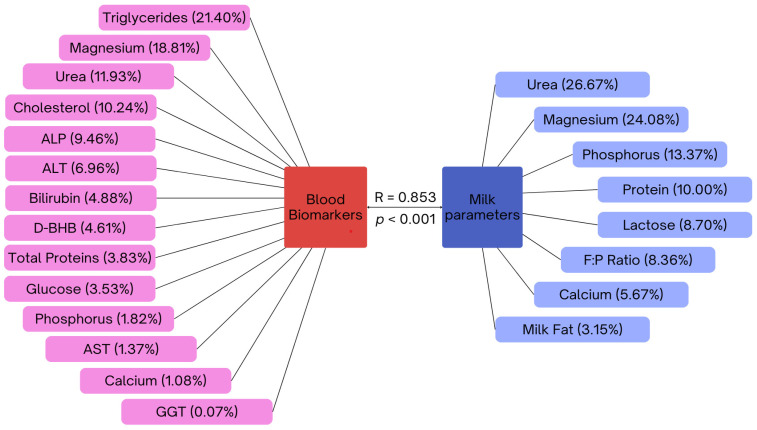
Percentual contributions (in brackets) of blood biomarkers (as canonical variate *U1*) and milk composition parameters (as canonical variate *V1*) to their respective canonical variates in the first canonical correlation (Table 6). The values in percentage correspond to the variable contributions to the selected canonical variate, based on the expression of canonical loading (data not presented) calculated as the absolute value of the loading for a specific variable divided by the sum of the absolute values of canonical loadings for all variables, multiplied by 100 for a specific canonical variable [38]. AST, aspartate aminotransferase; ALT, alanine aminotransferase; GGT, gamma-glutamyl transferase; ALP, alkaline phosphatase; D-BHB, D-beta-hydroxybutyrate; F:P ratio, milk fat to milk protein ratio.

Figure 1 presents percentage contribution values calculated from the canonical loading percentage, reflecting the variable contributions to the selected canonical variate. This is based on the formula: canonical loading (absolute value) divided by the sum of all canonical loadings (absolute value) multiplied by 100 for a specific canonical variable. Based on this data, we can rank the individual variables in the canonical variate U1 (blood biomarkers) as follows: TG > Mg > Urea > Chol > ALP > ALT > Bili > D-BHB > TP > Glu > P > AST > Ca > GGT. These findings suggest that TG, Mg, Urea, Chol, and ALP are the key biomarkers influencing canonical variation U1. Most milk variables demonstrated relatively high canonical loadings in the canonical variable V1, except for milk fat and Ca ranked as Urea > Mg > P > Protein > Lactose > F:P ratio > Ca > Milk Fat. Thus, milk urea, Mg, P, protein, and lactose significantly influence canonical variation V1, while Ca and milk fat contribute less.

## 4. Discussion

### 4.1. Blood Biomarkers of Dairy Cows

In the selected group of animals, we observed stable levels of liver enzymes (AST, ALT, ALP), glucose, bilirubin, and triglycerides. However, we noted increased levels of D-beta-hydroxybutyrate at the beginning of lactation. BHB is an important parameter related to the metabolic adaptation of dairy cows [1], potentially indicating a decrease in reproductive performance [9], reduced feed intake, or immune system disruption [39,40].

Furthermore, liver-related diseases present additional challenges that can significantly impact the metabolic health of dairy cows. These conditions not only impair overall health but also affect reproductive performance [2].

Interestingly, in our study, we did not observe elevated ALP activity, which is typically associated with liver, bone [41], or kidney [42,43] disorders. The average serum activities of other liver enzymes (AST and ALT) increased only slightly during lactation, with the highest values recorded during the PL stage. Notably, GGT activity was elevated throughout all stages of lactation compared to reference values [44]. The increased activities of AST and GGT may be linked to lower energy status [45], suggesting liver [46] and tissue damage often observed during energy deficit and ketosis [1,47].

In assessing metabolic health further, evaluation of the mineral profile is commonly used for screening metabolic disorders. The metabolism of calcium, phosphorus, and magnesium is facilitated through intestinal absorption, renal excretion, and exchanges in bones and soft tissue cells (particularly phosphorus and magnesium). Their concentration result from the action of physiological, pathophysiological, endogenous, and exogenous factors. A low serum calcium level (<2.0 mmol L^−1^) immediately post-calving serves as a risk indicator for subclinical hypocalcaemia [48]. In our study, serum calcium levels < 2.0 mmol L^−1^ were observed not only during the beginning of lactation but also at the peak and middle lactation stages. Additionally, serum mineral levels have been associated with peripartum diseases [49].

Moreover, phosphorus levels remained relatively balanced, with a slight increase in concentration observed at the end of lactation, although this change was not statistically significant. On the other hand, we found increased values of magnesium in the PL group, while the lowest values were recorded in the EL group. Fluctuations in mineral concentration may also be influenced by the calving system or by season, but without an obvious biological explanation [50].

In the individual groups of animals, we observed a slight increase in urea and total protein (TP) levels towards the end of lactation. However, the differences in cholesterol levels were statistically significant, aligning with findings from a study on Holstein Friesian and Simmental cows [51]. In their study, increased values were also noted in the middle of lactation, indicating that cholesterol plays a role in high milk production in this period [52].

### 4.2. Milk Composition During Different Lactation Stages and Their Relationship with Serum Biochemistry

As expected, milk yield values were highest in the peak of lactation (PL) group, gradually decreasing towards the end of lactation. Milk fat concentration varied non-significantly across lactation stages, decreasing from high levels at BL until ML and then increasing again until EL. Serum D-BHB levels revealed a similar trend, suggesting a link to subclinical ketosis.

This observation is critical since this metabolic disorder, associated with body fat mobilization, can lead to increased milk fat content [4,45]. We observed the highest D-BHB levels during BL, which coincided with the lowest GLU levels. Consistent with our findings, authors previously reported higher milk fat levels in metabolically burdened dairy cows (elevated D-BHB and decreased GLU levels) during the BL phase [45]. Elevated plasma BHB (Beta-hydroxybutyrate) levels, along with low plasma GLU levels, are commonly observed in dairy cows experiencing NEB during early lactation [11,39,53]. However, the correlations between these parameters and milk fat were not statistically significant in the present study.

Furthermore, results regarding serum CHOL and milk fat levels were consistent with our previous study [54]. Serum CHOL levels significantly influenced milk fat levels Reduced pH in the rumen (possibly pointing to SARA) triggers the release of lipopolysaccharides from the cell membranes of gram-negative bacteria [55], which interfere with lipid metabolism [56]. Rumen acidosis may result from the long-term consumption of fodder rich in easily fermentable saccharides [57]. This type of diet can also lead to a decrease in triglyceride levels [58].

According to Spearman’s correlation, milk protein content was the only milk composition parameter significantly associated with the lactation stage. It’s important to note that increased milk protein levels at EL may be attributed to hormonal changes associated with gravidity. It is well known that progesterone produced a few weeks after fertilization, impacts carbohydrate metabolism and promotes fat accumulation. Additionally, progesterone decreases gluconeogenesis [59], which may explain the higher protein content in milk during late lactation.

In our analysis, lactose emerged as the most stable component of milk, consistently above 4.8% throughout the lactation stages. This stability is a positive finding, as lower lactose concentrations might be a sign of mastitis and metabolic disorders in cows [60,61]. Similar trends in milk protein and lactose content have been documented in the Holstein breed [32].

Regarding nitrogen balance, milk urea level is a reliable indicator of nitrogen–energy balance in dairy cows [62]. Overfeeding with protein increases urea production and impacts milk quality [63,64]. In our study, the highest average urea level was observed during the BL and PL stages. In contrast, Konjačić et al. [62] reported the highest milk urea levels during the ML stage, while Yoon et al. [65] noted peak levels during the EL stage. These discrepancies suggested that the lactation phase may not significantly influence milk urea content. However, it should be noted that animal nutrition practices may vary across different countries.

Overall, Spearman’s and partial correlations showed positive associations between milk and serum urea levels. Highly positive correlations between these parameters have been reported in previous studies [66,67,68]. While glycaemia in the BL period was lower compared to EL, the opposite pattern was observed for milk urea content. This discrepancy may be connected to higher crude protein content in the PL period [69].

During periods of energy deficiency, rumen ammonia levels increase; this ammonia subsequently enters the bloodstream and is converted into urea by the liver [69]. Urea is then excreted from the body through all body fluids, including milk [62]. Thus, the significant positive correlation between serum ALT activity and milk urea level suggests an increased metabolic burden on the liver, also partly caused by increased intake of crude protein (in BL and PL).

In light of these metabolic factors, knowledge regarding macromineral metabolism in ruminants is relatively limited [70] despite the importance of these minerals in metabolism and overall health. Energy metabolism in dairy cows significantly impacts milk Mg and P levels, as both macrominerals are essential for ATP production via oxidative phosphorylation [71], as our results suggest according to partial correlation analysis.

Furthermore, as Spearman’s correlation analysis showed, milk calcium levels were significantly affected by blood triglyceride levels. The highest mean calcium concentration was measured at the beginning of lactation, probably due to hormonal activity associated with gravidity. Calcium and phosphorus are stored for fetal development and colostrum production [70] and are released into milk in greater quantities after calving. We found a significant negative impact of serum phosphorus level on milk calcium content. Additionally, milk calcium content was affected by increased activity of the liver enzyme ALT, which is diagnostically useful for assessing liver burden. However, the stage of lactation had no significant effect on the content of minerals in milk.

Since the fat-to-protein ratio is an alternative method for diagnosing subclinical ketosis, it positively correlated with blood BHB, cholesterol, and TG levels to our findings, previous studies have reported a positive relationship between BHB and the F:P ratio (*r* = 0.367), as well as between BHB and fat content (*r* = 0.265) [16], indicating that higher F:P ratios and BHB levels are associated with body reserves mobilization [72,73].

In this study, we also used Canonical Correlation Analysis (CCA) to analyze mutual associations among monitored variables by dividing them into two sets: blood biomarkers as the first set and milk composition parameters as the second. Identifying suitable biomarkers for monitoring changes in milk in response to the lactation phase or health status offers several options. The first canonical variable primarily reflected information about blood TG, while the second focused on milk urea. Based on the calculated associations through canonical loading, we identified significant groups of variables that play a decisive role in influencing each other. Specifically, key blood biomarkers include TG, Mg, urea, cholesterol, and ALP, while for milk composition parameters, urea, Mg, P, and protein are most relevant. Based on these findings, several key recommendations can be made based on the correlations found between milk composition and blood biomarkers, also considering the lactation stage. First of all, milk urea tends to be the most reliable biomarker connected to blood parameters in our set of animals. Attention should also be paid to monitoring optimal levels of minerals, in particular magnesium and phosphorus. In addition, understanding the fluctuations in milk protein, lactose levels, and F:P ratio can guide dietary strategies in all lactation stages.

## 5. Conclusions

Our findings revealed significant correlations between milk components and serum parameter levels, with notable differences observed across various lactation stages. Changes in milk composition can be influenced by multiple factors, and this study suggested key relationships between blood serum parameters and milk quality. Specifically, canonical correlation analysis identified three significant correlations, with blood triglycerides (TG) and milk urea being the strongest variables. The blood biomarkers associated with milk parameters included TG, magnesium, urea, cholesterol, and alkaline phosphatase, which correlated with milk urea, magnesium, phosphorus, protein, and lactose.

Given that milk composition fluctuates during different lactation stages, it is crucial for dairy farmers to monitor the nitrogen–energy balance and liver health in high-yielding dairy cows, as these factors are indicative of metabolic stress. Regular analysis of milk quality should be implemented as a routine practice to assess the overall health of the herd and identify potential metabolic issues early.

To improve the accuracy of interpretations regarding the relationships between milk and blood biomarkers, further research focused on specific lactation periods is needed. This ongoing investigation will help refine our understanding of these correlations, enabling more effective interventions to optimize dairy herd management and enhance milk production outcomes.

## Figures and Tables

**Table 1:** Feed composition during each stage of lactation (percentages as fed) and nutrient composition (percentages of dry matter).

**Item**	**BL**	**PL**	**ML**	**EL**
Corn silage (%)	34.0	36.0	37.0	41.0
Meadow Haylage (%)	34.0	35.0	36.0	40.0
Homemade mix (%)	17.0	17.5	15.5	-
High-moisture corn (%)	7.8	9.3	9.5	6.3
Cottonseed (%)	3.1	1.2	0.9	-
Wheat straw (%)	3.1	1.0	1.1	-
Mineral premix * (%)	1.0	-	-	0.7
Rapeseed scrap (%)	-	-	-	5.2
Oats grain (%)	-	-	-	5.1
DDGS ^1^ (%)	-	-	-	1.6
Magnesium (%)	-	-	-	0.07
**Nutrient Composition** **(% of the dry matter)**	**BL**	**PL**	**ML**	**EL**
Crude protein (%)	17.22	19.72	16.74	14.85
Crude fiber (%)	20.35	18.50	19.29	20.92
NDF ^2^ (%)	32.67	30.81	31.12	33.54
ADF ^3^ (%)	19.88	19.43	21.65	22.14
Crude fat (%)	4.30	5.89	4.03	3.36
NSC ^4^ (%)	36.12	40.83	39.13	32.11
Ash (%)	8.67	7.20	7.00	7.91
Calcium (%)	1.12	1.07	0.99	0.83
Phosphorus (%)	0.93	0.81	0.68	0.61
Sodium (%)	0.45	0.42	0.31	0.19
Potassium (%)	1.41	1.28	1.10	0.94
NEL ^5^ (MJ/kg)	6.86	6.69	6.56	6.21

^1^ DDGS—distiller’s dried grains with solubles; ^2^ NDF—neutral detergent fiber; ^3^ ADF—acid detergent fiber; ^4^ NSC—non-structural carbohydrates; ^5^ NEL—net energy for lactation; BL, beginning of lactation; PL, peak of lactation; ML, middle of lactation; EL, end of lactation. * 1 kg of mineral premix contains 18.0% Ca, 2.5% P, 9.0% Na, 8.0% Mg, 2000 mg Cu, 5000 mg Zn, 4500 mg Mn, 25 mg Co, 120 mg I, 35 mg Se, 700,000 U.I. Vitamin A, 180,000 U.I. Vitamin D3, 3000 mg Vitamin E, 80 mg Biotin.

**Table 2:** Intra- and inter-assay coefficients and sensitivity of selected biochemical parameters.

Parameter	Intra-Assay Coefficient (%)	Inter-Assay Coefficient (%)	Sensitivity
Ca	≤0.89	≤1.02	0.05 mmol L^−1^
P	≤1.20	≤1.53	0.065 mmol L^−1^
Mg	≤0.87	≤1.21	0.02 mmol L^−1^
UREA	≤1.91	≤2.04	0.33 mmol L^−1^
TP	≤0.90	≤1.30	0.05 g L^−1^
GLU	≤1.05	≤3.80	0.22 mmol L^−1^
AST	≤2.36	≤2.16	0.03 μkat L^−1^
ALT	≤2.90	≤2.05	0.07 μkat L^−1^
GGT	≤1.43	≤0.90	0.03 μkat L^−1^
ALP	≤1.16	≤1.10	0.05 μkat L^−1^
CHOL	≤0.95	≤1.10	0.08 mmol L^−1^
D-BHB	≤3.77	≤5.16	0.1 mmol L^−1^
BILI	≤2.11	≤3.03	1.2 μmol L^−1^
TG	≤1.61	≤1.23	0.01 mmol L^−1^

Ca, calcium; P, phosphorus; Mg, magnesium; TP, total protein; GLU, glucose; AST, aspartate aminotransferase; ALT, alanine aminotransferase; GGT, gamma-glutamyl transferase; ALP, alkaline phosphatase; CHOL, cholesterol; D-BHB, D-beta-hydroxybutyrate; BILI, bilirubin; TG, triglycerides.

**Table 3:** Serum parameters and milk composition of dairy cows during different lactation stages.

Parameter	Unit	BL		PL		ML		EL		*p* Value
		Mean	SEM	Mean	SEM	Mean	SEM	Mean	SEM	
**Serum Parameters**										
Ca	mmol L^−1^	1.98	0.09	1.91	0.11	1.82	0.11	2.15	0.09	0.151
P *	mmol L^−1^	1.80	0.10	1.90	0.13	1.94	0.08	2.22	0.17	0.220
Mg *	mmol L^−1^	0.96 ^ab^	0.05	1.05 ^a^	0.06	0.92 ^ab^	0.04	0.86 ^b^	0.03	0.016
UREA	mmol L^−1^	3.60	0.23	4.20	0.20	3.91	0.19	3.72	0.23	0.218
TP	g L^−1^	73.09	1.86	79.47	1.91	79.33	2.35	75.39	2.32	0.095
GLU	mmol L^−1^	3.33 ^b^	0.13	3.73 ^ab^	0.17	3.61 ^ab^	0.09	4.04 ^a^	0.13	0.004
AST *	µkat L^−1^	2.21	0.19	2.29	0.18	1.97	0.17	2.19	0.21	0.394
ALT *	µkat L^−1^	0.47	0.05	0.58	0.04	0.54	0.02	0.53	0.03	0.175
GGT *	µkat L^−1^	0.65 ^b^	0.06	0.90 ^a^	0.06	0.77 ^ab^	0.05	0.77 ^ab^	0.06	0.036
ALP	µkat L^−1^	1.44	0.14	1.64	0.11	1.56	0.12	1.87	0.16	0.145
CHOL	mmol L^−1^	3.21 ^c^	0.25	6.63 ^a^	0.36	5.27 ^b^	0.34	5.13 ^b^	0.35	0.001
D-BHB *	mmol L^−1^	0.79 ^a^	0.09	0.42 ^b^	0.03	0.46 ^b^	0.04	0.59 ^b^	0.03	0.002
BILI *	µmol L^−1^	15.68	0.61	15.17	0.59	15.93	0.52	14.10	0.87	0.332
TG *	mmol L^−1^	0.19	0.02	0.21	0.02	0.24	0.02	0.19	0.02	0.157
**Milk Composition/Performance**										
Ca	g L^−1^	1.36	0.07	1.25	0.05	1.34	0.09	1.29	0.03	0.599
P *	g L^−1^	0.92 ^ab^	0.05	0.77 ^b^	0.04	0.81 ^b^	0.03	0.89	0.02	0.004
Mg	mg L^−1^	87.57	3.87	90.04	3.19	90.96	3.97	84.13	3.03	0.550
Fat *	%	4.65	0.58	4.42	0.73	3.62	0.28	4.60	0.23	0.122
Protein *	%	3.32 ^b^	0.16	3.20 ^b^	0.09	3.37 ^b^	0.10	3.72 ^a^	0.08	0.001
Lactose *	%	4.80	0.05	4.86	0.05	4.87	0.05	4.85	0.06	0.552
Urea	mg dL^−1^	22.40	1.68	22.22	1.02	21.38	1.62	20.36	1.14	0.726
Milk yield	kg d^−1^	32.20 ^b^	0.76	38.26 ^a^	0.68	34.42 ^b^	0.70	33.02 ^b^	0.97	0.001
F:P ratio *		1.36	0.12	1.17	0.11	1.06	0.07	1.24	0.07	0.152

Data are expressed as means with pooled standard error of the mean (SEM). Mean values within a row with different superscripts differ significantly (*p* < 0.05). BL, beginning of lactation; PL, peak of lactation; ML, middle of lactation; EL, end of lactation; Ca, calcium; P, phosphorus; Mg, magnesium; TP, total protein; GLU, glucose; AST, aspartate aminotransferase; ALT, alanine aminotransferase; GGT, gamma-glutamyl transferase; ALP, alkaline phosphatase; CHOL, cholesterol; D-BHB, D-beta-hydroxybutyrate; BILI, bilirubin; TG, triglycerides; F:P ratio, milk fat to milk protein ratio; * marked parameters were analysed by non-parametric Kruskal-Wallis test with Dunn’s multiple comparisons.

**Table 4:** Spearman correlation coefficients (*r*) for the relationships between lactation stage, milk composition, and blood biomarkers.

Blood Biomarkers	Milk Composition Parameters								
	Lact. Stage	Ca	P	Mg	Fat	Protein	Lactose	Urea	F:P Ratio
Lact. stage		−0.0010	0.1058	−0.0687	0.0687	0.4209 *	0.1279	−0.0965	−0.0476
Ca	0.1026	0.2462 *	−0.0088	−0.3173 *	−0.0893	0.0424	0.2236 *	0.2255 *	−0.0337
P	0.2209 *	−0.5897 *	0.3905 *	−0.2180	0.0271	0.1864	0.2411 *	−0.4444 *	−0.0022
Mg	−0.2390 *	0.2990 *	−0.4156 *	0.4219 *	−0.2503 *	−0.3130 *	0.1191	0.1922	−0.1661
UREA	−0.0064	0.1775	−0.2967 *	−0.1396	−0.1350	−0.1682	0.1352	0.5643 *	−0.0913
TP	0.0867	−0.0713	−0.2394 *	0.1700	−0.1470	0.0008	−0.0978	0.1626	−0.2518 *
GLU	0.3375 *	0.1205	0.1718	−0.1990	0.0653	0.1816	−0.0247	0.1630	−0.0347
AST	−0.0869	0.1058	−0.0778	0.0084	0.0111	−0.0402	0.0676	−0.0039	−0.0476
ALT	0.1411	0.4896 *	−0.2965 *	0.1002	−0.1297	0.0114	0.1371	0.4054 *	−0.0657
GGT	0.0880	−0.1505	−0.1221	−0.0952	−0.2538 *	−0.1284	0.2866 *	−0.1503	−0.1873
ALP	0.2159	−0.1020	0.3140 *	−0.0244	0.0058	0.1600	0.1553	−0.3740 *	0.0011
CHOL	0.2834 *	0.0584	−0.2708 *	0.0372	−0.2419 *	−0.0335	0.4424 *	0.1432	−0.2177 *
D-BHB	−0.1274	−0.0898	0.1904	−0.2508 *	0.1811	0.1392	0.0421	−0.0719	0.2344 *
BILI	−0.0957	−0.2332	0.0224	0.0853	0.1146	0.0976	−0.1956	−0.2277 *	0.0468
TG	0.0320	−0.3769 *	−0.0034	0.3161 *	0.1163	−0.0006	−0.1118	−0.4667 *	0.0359

* *p* < 0.05; Lact. Stage, lactation stage; Ca, calcium; P, phosphorus; Mg, magnesium; TP, total protein; GLU, glucose; AST, aspartate aminotransferase; ALT, alanine aminotransferase; GGT, gamma-glutamyl transferase; ALP, alkaline phosphatase; CHOL, cholesterol; D-BHB, D-beta-hydroxybutyrate; BILI, bilirubin; TG, triglycerides; F:P ratio, milk fat to milk protein ratio.

**Table 5:** Partial correlation coefficients (*r*) for the relationship between milk composition versus blood biomarkers after including the effect of lactation stage.

Blood Biomarkers	Milk Composition Parameters							
	Ca	P	Mg	Fat	Protein	Lactose	Urea	F:P Ratio
Ca	0.3713 *	−0.1399	−0.1577	−0.0048	0.1384	0.1366	−0.1828	−0.0951
P	−0.4620 *	0.2871 *	0.0733	−0.3220 *	−0.1787	−0.3324 *	0.1620	0.2735 *
Mg	−0.1031	−0.4030 *	0.5316 *	0.0886	−0.1027	0.1465	−0.1466	0.0967
UREA	−0.0787	0.0418	−0.0239	0.2256	−0.1321	0.0141	0.4569 *	−0.0807
TP	−0.1827	0.0469	0.0033	−0.0415	−0.0289	−0.1788	0.0595	−0.1123
GLU	−0.2571	0.2062	−0.0462	0.1482	−0.0706	−0.1480	0.1243	−0.0215
AST	0.1006	−0.0788	−0.0595	0.0342	0.1044	0.0889	−0.1774	0.0751
ALT	0.1705	−0.1904	0.1025	0.0279	0.0456	0.0622	0.1539	−0.0914
GGT	−0.0623	−0.0719	−0.0672	−0.0814	0.0030	−0.0143	0.0031	−0.0366
ALP	0.0783	0.0033	0.1299	−0.0760	−0.0602	0.1095	−0.2997 *	0.0519
CHOL	−0.1350	−0.3327 *	0.2539	0.1817	0.0588	0.3445 *	−0.1415	−0.0653
D-BHB	−0.0976	0.1579	−0.2221	0.0410	0.0944	−0.0524	0.0714	0.0820
BILI	0.1296	−0.0028	−0.1063	0.0647	0.1604	−0.1195	−0.0670	−0.2326
TG	−0.0791	−0.3478 *	0.3756 *	0.2333	−0.0769	0.2820 *	−0.1562	0.0191

* *p* < 0.05; Ca, calcium; P, phosphorus; Mg, magnesium; TP, total protein; GLU, glucose; AST, aspartate aminotransferase; ALT, alanine aminotransferase; GGT, gamma-glutamyl transferase; ALP, alkaline phosphatase; CHOL, cholesterol; D-BHB, D-beta-hydroxybutyrate; BILI, bilirubin; TG, triglycerides; F:P ratio, milk fat to milk protein ratio.

**Table 6:** Canonical correlations (*r_c_*) between blood biomarkers and milk composition parameters (* *p* < 0.05).

Number of Canonical Function	Canonical Correlation	Eigenvalue	Percentage of Variation	*p* Value
1	0.853	2.672	0.363	0.000 *
2	0.823	2.097	0.285	0.000 *
3	0.739	1.202	0.163	0.002 *
4	0.602	0.567	0.077	0.133
5	0.554	0.443	0.060	0.428
6	0.441	0.242	0.033	0.836
7	0.285	0.088	0.012	0.964
8	0.209	0.046	0.006	0.921

## Data Availability

The data presented in this study are available upon reasonable request from the corresponding author.

## References

[1] Kessel S., Stroehl M., Meyer H.H.D., Hiss S., Sauerwein H., Schwarz F.J., Bruckmaier R.M. (2008). Individual Variability in Physiological Adaptation to Metabolic Stress during Early Lactation in Dairy Cows Kept under Equal Conditions. J. Anim. Sci..

[2] Ringseis R., Gessner D.K., Eder K. (2015). Molecular Insights into the Mechanisms of Liver-Associated Diseases in Early-Lactating Dairy Cows: Hypothetical Role of Endoplasmic Reticulum Stress. J. Anim. Physiol. Anim. Nutr..

[3] Sundrum A. (2015). Metabolic Disorders in the Transition Period Indicate That the Dairy Cows’ Ability to Adapt Is Overstressed. Animals.

[4] Giannuzzi D., Toscano A., Pegolo S., Gallo L., Tagliapietra F., Mele M., Minuti A., Trevisi E., Ajmone Marsan P., Schiavon S. (2022). Associations between Milk Fatty Acid Profile and Body Condition Score, Ultrasound Hepatic Measurements and Blood Metabolites in Holstein Cows. Animals.

[5] Graber M., Kohler S., Müller A., Burgermeister K., Kaufmann T., Bruckmaier R.M., van Dorland H.A. (2012). Identification of Plasma and Hepatic Parameters Related to Metabolic Robustness in Dairy Cows: Plasma and Hepatic Parameters Describing Robustness. J. Anim. Physiol. Anim. Nutr..

[6] Loor J.J., Everts R.E., Bionaz M., Dann H.M., Morin D.E., Oliveira R., Rodriguez-Zas S.L., Drackley J.K., Lewin H.A. (2007). Nutrition-Induced Ketosis Alters Metabolic and Signaling Gene Networks in Liver of Periparturient Dairy Cows. Physiol. Genom..

[7] McCarthy S.D., Waters S.M., Kenny D.A., Diskin M.G., Fitzpatrick R., Patton J., Wathes D.C., Morris D.G. (2010). Negative Energy Balance and Hepatic Gene Expression Patterns in High-Yielding Dairy Cows during the Early Postpartum Period: A Global Approach. Physiol. Genom..

[8] Vallejo-Timarán D., Reyes-Vélez J., VanLeeuwen J., Maldonado-Estrada J., Astaiza-Martínez J. (2020). Incidence and Effects of Subacute Ruminal Acidosis and Subclinical Ketosis with Respect to Postpartum Anestrus in Grazing Dairy Cows. Heliyon.

[9] Alemu T.W., Santschi D.E., Cue R.I., Duggavathi R. (2023). Reproductive Performance of Lactating Dairy Cows with Elevated Milk β-Hydroxybutyrate Levels during First 6 Weeks of Lactation. J. Dairy Sci..

[10] Leblanc S. (2010). Monitoring Metabolic Health of Dairy Cattle in the Transition Period. J. Reprod. Dev..

[11] Hammon H.M., Stürmer G., Schneider F., Tuchscherer A., Blum H., Engelhard T., Genzel A., Staufenbiel R., Kanitz W. (2009). Performance and Metabolic and Endocrine Changes with Emphasis on Glucose Metabolism in High-Yielding Dairy Cows with High and Low Fat Content in Liver after Calving. J. Dairy Sci..

[12] Stengärde L., Tråvén M., Emanuelson U., Holtenius K., Hultgren J., Niskanen R. (2008). Metabolic Profiles in Five High-Producing Swedish Dairy Herds with a History of Abomasal Displacement and Ketosis. Acta Vet. Scand..

[13] Walter L.L., Gärtner T., Gernand E., Wehrend A., Donat K. (2022). Effects of Parity and Stage of Lactation on Trend and Variability of Metabolic Markers in Dairy Cows. Animals.

[14] Andrews A.H. (2000). The Health of Dairy Cattle.

[15] Ježek J., Cincović M.R., Nemec M., Belić B., Djoković R., Klinkon M., Starič J. (2017). Beta-Hydroxybutyrate in Milk as Screening Test for Subclinical Ketosis in Dairy Cows. Pol. J. Vet. Sci..

[16] Glatz-Hoppe J., Boldt A., Spiekers H., Mohr E., Losand B. (2020). Relationship between Milk Constituents from Milk Testing and Health, Feeding, and Metabolic Data of Dairy Cows. J. Dairy Sci..

[17] Churakov M., Karlsson J., Edvardsson Rasmussen A., Holtenius K. (2021). Milk Fatty Acids as Indicators of Negative Energy Balance of Dairy Cows in Early Lactation. Animal.

[18] Jenkins N.T., Peña G., Risco C., Barbosa C.C., Vieira-Neto A., Galvão K.N. (2015). Utility of Inline Milk Fat and Protein Ratio to Diagnose Subclinical Ketosis and to Assign Propylene Glycol Treatment in Lactating Dairy Cows. Can. Vet. J. Rev. Veterinaire Can..

[19] Tremblay M., Kammer M., Lange H., Plattner S., Baumgartner C., Stegeman J.A., Duda J., Mansfeld R., Döpfer D. (2018). Identifying Poor Metabolic Adaptation during Early Lactation in Dairy Cows Using Cluster Analysis. J. Dairy Sci..

[20] Mills S., Ross R.P., Hill C., Fitzgerald G.F., Stanton C. (2011). Milk Intelligence: Mining Milk for Bioactive Substances Associated with Human Health. Int. Dairy J..

[21] Osorio J.S., Trevisi E., Ji P., Drackley J.K., Luchini D., Bertoni G., Loor J.J. (2014). Biomarkers of Inflammation, Metabolism, and Oxidative Stress in Blood, Liver, and Milk Reveal a Better Immunometabolic Status in Peripartal Cows Supplemented with Smartamine M or MetaSmart. J. Dairy Sci..

[22] Bland J.H., Grandison A.S., Fagan C.C. (2015). Evaluation of Milk Compositional Variables on Coagulation Properties Using Partial Least Squares. J. Dairy Res..

[23] Canive M., Casais R., Jimenez J.A., Blanco-Vazquez C., Amado J., Garrido J.M., Juste R.A., Alonso-Hearn M. (2020). Correlations between Single Nucleotide Polymorphisms in Bovine CD209, SLC11A1, SP110 and TLR2 Genes and Estimated Breeding Values for Several Traits in Spanish Holstein Cattle. Heliyon.

[24] Leitner G., Krifucks O., Merin U., Lavi Y., Silanikove N. (2006). Interactions between Bacteria Type, Proteolysis of Casein and Physico-Chemical Properties of Bovine Milk. Int. Dairy J..

[25] Martí-De Olives A., Le Roux Y., Rubert-Alemán J., Peris C., Molina M.P. (2011). Short Communication: Effect of Subclinical Mastitis on Proteolysis in Ovine Milk. J. Dairy Sci..

[26] Miluchová M., Gábor M., Candrák J. (2023). The Effect of the Genotypes of the CSN2 Gene on Test-Day Milk Yields in the Slovak Holstein Cow. Agriculture.

[27] Silanikove N., Leitner G., Merin U., Prosser C.G. (2010). Recent Advances in Exploiting Goat’s Milk: Quality, Safety and Production Aspects. Small Rumin. Res..

[28] Sundekilde U.K., Frederiksen P.D., Clausen M.R., Larsen L.B., Bertram H.C. (2011). Relationship between the Metabolite Profile and Technological Properties of Bovine Milk from Two Dairy Breeds Elucidated by NMR-Based Metabolomics. J. Agric. Food Chem..

[29] Wang E., Cha M., Wang S., Wang Q., Wang Y., Li S., Wang W. (2023). Feeding Corn Silage or Grass Hay as Sole Dietary Forage Sources: Overall Mechanism of Forages Regulating Health-Promoting Fatty Acid Status in Milk of Dairy Cows. Foods.

[30] Cashman K.D. (2006). Milk Minerals (Including Trace Elements) and Bone Health. Int. Dairy J..

[31] van Knegsel A.T.M., Mollenhorst H., Goselink R.M.A., de Haas Y. (2020). Milk Analysis and Cow Health: Predicting Dairy Cow Life Span with Milk Sampling in Early Lactation.

[32] Andjelić B., Djoković R., Cincović M., Bogosavljević-Bošković S., Petrović M., Mladenović J., Čukić A. (2022). Relationships between Milk and Blood Biochemical Parameters and Metabolic Status in Dairy Cows during Lactation. Metabolites.

[33] NRC (National Research Council) (2001). Nutrient Requirements of Dairy Cattle.

[34] Rolinec M., Bíro D., Šimko M., Juráček M., Hanušovský O., Schubertová Z., Chadimová L., Gálik B. (2021). Grape Pomace Ingestion by Dry Cows Does Not Affect the Colostrum Nutrient and Fatty Acid Composition. Animals.

[35] Juráček M., Vašeková P., Massányi P., Kováčik A., Bíro D., Šimko M., Gálik B., Rolinec M., Hanušovský O., Kolláthová R. (2021). The Effect of Dried Grape Pomace Feeding on Nutrients Digestibility and Serum Biochemical Profile of Wethers. Agriculture.

[36] Kovacik A., Arvay J., Tusimova E., Harangozo L., Tvrda E., Zbynovska K., Cupka P., Andrascikova S., Tomas J., Massanyi P. (2017). Seasonal Variations in the Blood Concentration of Selected Heavy Metals in Sheep and Their Effects on the Biochemical and Hematological Parameters. Chemosphere.

[37] Kovacikova E., Kovacik A., Halenar M., Tokarova K., Chrastinova L., Ondruska L., Jurcik R., Kolesar E., Valuch J., Kolesarova A. (2019). Potential Toxicity of Cyanogenic Glycoside Amygdalin and Bitter Apricot Seed in Rabbits-Health Status Evaluation. J. Anim. Physiol. Anim. Nutr..

[38] Vargas J.A.C., Botelho Duarte Gomes V.D.S., Mezzomo R., Maciel R.P. (2023). Multivariate Relationship between Major Constituents and Casein Fractions in Buffalo Milk Using Canonical Correlation Analysis. Int. Dairy J..

[39] Gross J., van Dorland H.A., Bruckmaier R.M., Schwarz F.J. (2011). Performance and Metabolic Profile of Dairy Cows during a Lactational and Deliberately Induced Negative Energy Balance with Subsequent Realimentation. J. Dairy Sci..

[40] Horst E.A., Kvidera S.K., Baumgard L.H. (2021). Invited Review: The Influence of Immune Activation on Transition Cow Health and Performance—A Critical Evaluation of Traditional Dogmas. J. Dairy Sci..

[41] Garnero P., Delmas P.D. (1993). Assessment of the Serum Levels of Bone Alkaline Phosphatase with a New Immunoradiometric Assay in Patients with Metabolic Bone Disease. J. Clin. Endocrinol. Metab..

[42] Beddhu S., Ma X., Baird B., Cheung A.K., Greene T. (2009). Serum Alkaline Phosphatase and Mortality in African Americans with Chronic Kidney Disease. Clin. J. Am. Soc. Nephrol..

[43] Peters E., Heemskerk S., Masereeuw R., Pickkers P. (2014). Alkaline Phosphatase: A Possible Treatment for Sepsis-Associated Acute Kidney Injury in Critically Ill Patients. Am. J. Kidney Dis..

[44] MSD Veterinary Manual Serum Biochemical Analysis Reference Ranges—Special Subjects. https://www.msdvetmanual.com/special-subjects/reference-guides/serum-biochemical-analysis-reference-ranges.

[45] van Dorland H.A., Graber M., Kohler S., Steiner A., Bruckmaier R.M. (2014). Comparison of Hepatic Adaptation in Extreme Metabolic Phenotypes Observed in Early Lactation Dairy Cows On-Farm. J. Anim. Physiol. Anim. Nutr..

[46] Moore F. (1997). Interpreting Serum Chemistry Profiles in Dairy Cows. Vet. Med. 1985 USA.

[47] Mills S.E., Beitz D.C., Young J.W. (1986). Characterization of Metabolic Changes during a Protocol for Inducing Lactation Ketosis in Dairy Cows. J. Dairy Sci..

[48] Oetzel G.R. (2004). Monitoring and Testing Dairy Herds for Metabolic Disease. Vet. Clin. N. Am. Food Anim. Pract..

[49] Van Saun R.J., Todd A., Varga G.A. Serum Mineral Status and Risk of Periparturient Disease. Proceedings of the XXIV World Buiatrics Congress.

[50] Brscic M., Cozzi G., Lora I., Stefani A.L., Contiero B., Ravarotto L., Gottardo F. (2015). Short Communication: Reference Limits for Blood Analytes in Holstein Late-Pregnant Heifers and Dry Cows: Effects of Parity, Days Relative to Calving, and Season. J. Dairy Sci..

[51] Obućinski D., Soleša D., Kučević D., Prodanović R., Tomaš Simin M., Ljubojević Pelić D., Đuragić O., Puvača N. (2019). Management of Blood Lipid Profile and Oxidative Status in Holstein and Simmental Dairy Cows during Lactation. Mljekarstvo.

[52] Kessler E.C., Gross J.J., Bruckmaier R.M., Albrecht C. (2014). Cholesterol Metabolism, Transport, and Hepatic Regulation in Dairy Cows during Transition and Early Lactation. J. Dairy Sci..

[53] Van Q.C.D., Knapp E., Hornick J.-L., Dufrasne I. (2020). Influence of Days in Milk and Parity on Milk and Blood Fatty Acid Concentrations, Blood Metabolites and Hormones in Early Lactation Holstein Cows. Animals.

[54] Tušimová E., Kováčik A., Harangozo Ľ., Lukáč N., Kolesárová A., Vollmannová A., Kováčik J. (2015). Internal Milieau of Dairy Cows at the Beginning of Lactation and Its Influence on Composition of Raw Milk. J. Microbiol. Biotechnol. Food Sci..

[55] Emmanuel D.G.V., Dunn S.M., Ametaj B.N. (2008). Feeding High Proportions of Barley Grain Stimulates an Inflammatory Response in Dairy Cows. J. Dairy Sci..

[56] Feingold K., Staprans I., Memon R., Moser A., Shigenaga J., Doerrler W., Dinarello C., Grunfeld C. (1992). Endotoxin Rapidly Induces Changes in Lipid Metabolism That Produce Hypertriglyceridemia: Low Doses Stimulate Hepatic Triglyceride Production While High Doses Inhibit Clearance. J. Lipid Res..

[57] Gozho G.N., Plaizier J.C., Krause D.O., Kennedy A.D., Wittenberg K.M. (2005). Subacute Ruminal Acidosis Induces Ruminal Lipopolysaccharide Endotoxin Release and Triggers an Inflammatory Response. J. Dairy Sci..

[58] Xu T., Tao H., Chang G., Zhang K., Xu L., Shen X. (2015). Lipopolysaccharide Derived from the Rumen Down-Regulates Stearoyl-CoA Desaturase 1 Expression and Alters Fatty Acid Composition in the Liver of Dairy Cows Fed a High-Concentrate Diet. BMC Vet. Res..

[59] Masuyama H., Hiramatsu Y. (2011). Potential Role of Estradiol and Progesterone in Insulin Resistance through Constitutive Androstane Receptor. J. Mol. Endocrinol..

[60] Costa A., Lopez-Villalobos N., Sneddon N.W., Shalloo L., Franzoi M., De Marchi M., Penasa M. (2019). Invited Review: Milk Lactose—Current Status and Future Challenges in Dairy Cattle. J. Dairy Sci..

[61] Pyörälä S. (2003). Indicators of Inflammation in the Diagnosis of Mastitis. Vet. Res..

[62] Konjačić M., Kelava N., Ivkić Z., Ivanković A., Prpić Z., Vnučec I., Ramljak J., Mijić P. (2010). Non-Nutritional Factors of Milk Urea Concentration in Holstein Cows from Large Dairy Farms in Croatia. Mljekarstvo.

[63] Frank B., Swensson C. (2002). Relationship Between Content of Crude Protein in Rations for Dairy Cows and Milk Yield, Concentration of Urea in Milk and Ammonia Emissions. J. Dairy Sci..

[64] Kováčik J., Kalafová A., Tušimová E. (2013). Relations between Selected Indicators of Blood and Milk of Dairy Cows with Metabolic Disorders. J. Microbiol. Biotechnol. Food Sci..

[65] Yoon J.T., Lee J.H., Kim C.K., Chung Y.C., Kim C.-H. (2004). Effects of Milk Production, Season, Parity and Lactation Period on Variations of Milk Urea Nitrogen Concentration and Milk Components of Holstein Dairy Cows. Asian-Australas. J. Anim. Sci..

[66] Butler W.R., Calaman J.J., Beam S.W. (1996). Plasma and Milk Urea Nitrogen in Relation to Pregnancy Rate in Lactating Dairy Cattle. J. Anim. Sci..

[67] Broderick G.A., Clayton M.K. (1997). A Statistical Evaluation of Animal and Nutritional Factors Influencing Concentrations of Milk Urea Nitrogen. J. Dairy Sci..

[68] Campanile G., De Filippo C., Di Palo R., Taccone W., Zicarelli L. (1998). Influence of Dietary Protein on Urea Levels in Blood and Milk of Buffalo Cows. Livest. Prod. Sci..

[69] Bendelja Ljoljić D., Dolenčić Špehar I., Prpić Z., Vnučec I., Samaržija D. (2020). Urea Concentration in Goat Milk: Importance of Determination and Factors of Variability. J. Cent. Eur. Agric..

[70] Härter C.J., Castagnino D.S., Rivera A.R., Lima L.D., Silva H.G.O., Mendonça A.N., Bonfim G.F., Liesegang A., St-Pierre N., Teixeira I.A.M.A. (2015). Mineral Metabolism in Singleton and Twin-Pregnant Dairy Goats. Asian-Australas. J. Anim. Sci..

[71] Lima L.D., Kozloski G.V., Bonnecarrère Sanchez L.M., Ruggia Chiesa A.P., Härter C.J., Fiorentini G., Oliveira L., Cadorin R.L. (2008). Effect of Harvesting Period on the Nutritive Value of Rice Grass (*Echinochloa* sp.) Hay given as Sole Diet to Lambs. Small Rumin. Res..

[72] Heuer C., Van Straalen W.M., Schukken Y.H., Dirkzwager A., Noordhuizen J.P.T.M. (2000). Prediction of Energy Balance in a High Yielding Dairy Herd in Early Lactation: Model Development and Precision. Livest. Prod. Sci..

[73] Nielsen N.I., Ingvartsen K.L., Larsen T. (2003). Diurnal Variation and the Effect of Feed Restriction on Plasma and Milk Metabolites in TMR-fed Dairy Cows. J. Vet. Med. Ser. A.

